# Voice Endorsement and Employee Safety Voice Behavior in Construction Projects: The Mediating Role of Leader-Member Exchange

**DOI:** 10.3390/ijerph19063374

**Published:** 2022-03-13

**Authors:** Yunfeng Sun, Hao Yang, Chongyang Qian, Yifeng Jiang, Xiaowei Luo, Xiang Wu

**Affiliations:** 1 School of Engineering and Technology, China University of Geosciences (Beijing), Beijing 100083, China; 2002200097@cugb.edu.cn (Y.S.); wuxiang@cugb.edu.cn (X.W.); 2 Institute of Urban Safety and Environmental Science, Beijing Academy of Science and Technology, Beijing 100054, China; 15201018237@163.com; 3 China Electric Power Research Institute, Beijing 100192, China; jiangyifeng@epri.sgcc.com.cn; 4 Department of Architecture and Civil Engineering, City University of Hong Kong, Hong Kong 999077, China; xiaowluo@cityu.edu.hk

**Keywords:** construction projects, voice endorsement, leader-member exchange, employee safety voice behavior

## Abstract

Employee safety voice refers to publishing opinions and suggestions related to workplace safety issues. In recent years, it has gradually become a hot topic in the field of organizational safety management research. Voice endorsement is the leader’s positive feedback to employees, and it is a necessary condition and key link for employees to achieve the purpose of voicing. Although there are many types of research on employee safety voice behavior and voice endorsement, few studies have explored the relationship between the two. Therefore, through a paired questionnaire survey of 214 leaders and 344 employees in construction projects, drawing on social exchange theory, using leader-member exchange (LMX) as a mediating variable, we discuss the mechanism of voice endorsement on employee safety voice behavior. The results show that in construction projects, voice endorsement negatively affects employee safety voice behavior and LMX, and LMX positively affects employee safety voice behavior. LMX has a mediating role in the relationship between voice endorsement and employee safety voice behavior. The results of this study can provide useful guidance for improving employee safety voice behavior management.

## 1. Introduction

In construction projects, accidents occur frequently, which seriously affects the safety of employees and is detrimental to the long-term development of the organization [1]. Employee safety voice is significant for preventing accidents and improving the safety performance of the organization [2]. In the context of work safety, Tucker and Chmiel [3] defined employee safety voice as “communications designed to change the perceived unsafe working conditions that have an impact on the health of individuals and organizations.” They argued that the purpose of safety voice is to improve the safety of the working environment (for example, to promote actions to establish safer procedures), and it is challenging to the status quo [3]. As a special type of voice, employee safety voice helps organizations find safety problems and take measures to avoid accidents [4]. Employees’ opinions and suggestions related to work safety are significant to the survival and development of organizations, thus safety voice has become an increasingly popular research topic [5,6].

Employees usually put forward safety voice to leaders, so leadership behavior is an important factor that affects employee safety voice [3,7,8]. Although some scholars indicated that leaders actively endorsing employee voice can promote employee safety voice [3], few studies have explored in detail the mechanism of voice endorsement on employee safety voice. Voice endorsement represents the degree of attention and recognition of the leaders to the suggestions and opinions put forward by subordinates [9]. It is a good way for superiors to give feedback to their subordinates and an important way for employees to promote the organization [10], that is, voice endorsement represents leaders recognition and support for employees [11], and it is a supportive resource provided by organizations and leaders to employees. In return, when voice is endorsed, employees will work hard to help the organization achieve its work goals [12]. Wu and Kee [13] found that voice endorsement can foster employee voice through the mediating effects of positive emotion and work engagement. Safety voice is very different from employee voice. Safety voice is more concerned with safety than employee voice. A lack of safety voice can lead to serious accidents, and safety voice is more significant in reducing risk in the workplace [8]. Few previous studies have focused on how voice endorsement plays a role in safety voice, so the mechanisms by which voice endorsement influences employee safety voice behavior need to be explored.

Leader–member exchange (LMX) is an important leadership factor that affects the behaviors of employees. The core of LMX theory is that leaders can form different LMX relationships with employees through long-term interaction with their subordinates. LMX quality affects employees’ perceptions of their leaders, which in turn affects their work behaviors [14,15]. Previous studies mostly explored the relationship between safety voice-related concepts and LMX. By building a good LMX relationship and providing more diversified organizational support, employees can feel more responsibilities and obligations to participate in the improvement of workplace safety [16,17]. In high-quality LMX, employees have a lot of communication and exchange with leaders, and a good relationship will be built between them, and employees will receive special care and support from leaders [18,19,20]. Since employees with high-quality LMX relationship can get more trust and support from the leaders, they have more courage to conduct safety voice behavior. At present, many studies have confirmed the promotion effect of leadership support on employee safety voice [2,3]. However, few studies have focused on the influence mechanism of LMX on employee safety voice behavior. 

Therefore, this research tries to explore the mechanism of leader’s voice endorsement on employee safety voice behavior, and the mediating role of LMX between voice endorsement and employee safety voice behavior in construction projects. Through the empirical test, the conclusion of this study will help to further investigate the mechanism of employee safety voice behavior and provide theoretical reference, so as to promote enterprise leaders to endorse employee safety voice effectively and ultimately improve the level of safety management and decision-making.

## 2. Literature Review and Hypotheses Development

### 2.1. Voice Endorsement and Employee Safety Voice

Graen and Uhl-Bien [21] pointed out that in the study of the relationship between leaders and employees, a key theoretical basis is the social exchange theory based on the principle of equality and reciprocity. The social exchange theory was first put forward in the microeconomic research related to human behavior. Social exchange theory is an important theory to explain human behavior. The basic idea is that human beings are driven by self-interest to make reciprocal exchanges with others in order to satisfy their needs, and in the process of exchange, they strive to maximize their interests. Its essence is that in the two parties with interests, one party uses actual actions to return the beneficial swap behavior of the other party [22]. After social exchange theory was introduced into the field of organizational management, many scholars found that social exchange theory can explain the interaction between leaders and employees [23,24,25]. In the process of social interaction, leaders and subordinates will respect each other; generate trust and reward each other’s sense of obligation; and then influence, support, and share resources with each other [26].

Safety voice usually refers to the act of raising concerns about safety issues through formal or informal channels [3], which can provide people with an opportunity to communicate the safety situation and ultimately make people form a common understanding of risks, so as to take corresponding measures to reduce the risk [27]. Employee safety voice behavior is an important factor to reduce danger [28]. Voice endorsement refers to whether the leaders will be interested in the suggestions and willing to support the subordinates when the subordinates put forward their opinions or suggestions on the aspects of the organization [9]. Therefore, the leaders’ voice endorsement represents their recognition and support for the employees who put forward safety suggestions. Moreover, the leaders can represent the organization [29], so employees will perceive the trust and support of the whole organization through the leaders’ support behaviors. According to the reciprocity criterion of social exchange theory, when employees feel the trust and support from the organization or leaders, they will have a sense of obligation to repay the organization and the leaders, make some return behavior, and even be willing to sacrifice their interests for the development of the organization [30]. The implementation of extra-role behavior is a way for subordinates to repay their leaders [31]. Safety voice is an extra-role behavior [32]. Therefore, employees are more likely to make safety voice when they return to organizations and leaders. Accordingly, the following hypothesis is proposed:

**Hypothesis** **1.** *Voice endorsement positively influences employee safety voice behavior*.

### 2.2. The Mediating Role of LMX

To better explain the influence mechanism of voice endorsement on employee safety voice, we introduce LMX as a mediating variable. The core content of LMX theory is that leaders cannot treat every member of the team equally [33,34]. Graen and Uhl Bien [21] argued that employees with high-quality LMX have access to more resources, information, as well as better prospective work assignments. On the contrary, low-quality LMX employees have difficulty in obtaining resource support from their leaders and only complete their work assignments according to their job responsibilities, making it difficult to achieve outstanding performance and status.

At present, the social exchange theory based on the principle of reciprocity has become the core theory to explain the exchange relationship between leaders and members. According to the theory, high-quality LMX employees are given more resource support and information sharing than low-quality LMX employees so that the employees can master more organizational resources; the closeness of the relationship with the leader, in turn, affects the performance of the employees, in exchange, high-quality LMX employees are dispatched to work with higher motivation, thus showing a more engaged work status and better organizational citizenship behavior [23,35,36].

Some studies have shown that leadership behavior has an important impact on LMX quality [37,38,39]. The influence of voice endorsement on LMX can be explained from two aspects: First, voice endorsement helps employees to judge whether their cognition of organizational problems is accurate through leader’s supportive attitude so as to reduce organizational conflicts caused by individual role ambiguity, thus forming a high-quality LMX relationship; second, the social exchange theory holds that reciprocal norms are the core principle of LMX, and the mutual feedback based on each other’s contribution is the basis for the maintenance and development of the exchange relationship [40,41]. When individuals get the benefits provided by others, they will correspondingly give back the necessary benefits to the benefit providers. These kinds of reciprocal norms will develop towards mutual trust and positive state, and eventually both sides will establish high-quality social exchange relations [42]. Voice endorsement will make employees feel the trust of leaders. In return, they will also have more trust in their superiors and think that the relationship between themselves and their superiors is mutual respect and satisfaction, which is conducive to the formation of high-quality LMX relations. From the perspective of social exchange, Xia et al. argued that the employees whose opinions were adopted could feel the support of the leaders and show the behaviors of rewarding the leaders. Therefore, LMX would improve [43]. Xia et al. also confirmed the positive effect of voice endorsement on LMX through empirical research [43]. Accordingly, the following hypothesis is proposed:

**Hypothesis** **2.** *Voice endorsement positively influences LMX*.

Safety voice behavior often means dissatisfaction with the current corporate safety system and challenges to the authority of the leaders, so it has a certain degree of risk, and employees will feel scared when expressing their safety concerns [6,8]. When the quality of the LMX relationship is high, employees will get the opportunity for respect, trust, and promotion provided by the leaders, and a good atmosphere will be formed between the leader and the employee [21,36], thereby reducing the fear of employees making safety suggestions. In addition, the process of social exchange is a process of individual decision making, and the principle of individual decision making includes the principle of altruism in addition to the principle of reciprocity. The principle of altruism means both parties to an exchange will try to satisfy each other’s needs even if it may harm their own interests. The theory of social exchange shows that in a high-quality LMX relationship, employees will show a more serious and responsible attitude in their work in return for leaders’ favor. Therefore, employees may reward their leaders through safety voice, which is conducive to organizational safety. Many studies have shown that LMX has an important impact on employee behavior [44,45,46]. Accordingly, the following hypothesis is proposed:

**Hypothesis** **3.** *LMX positively influences employee safety voice behavior*.

As mentioned above, voice endorsement, as a supportive resource, represents leadership’s recognition and encouragement of employees’ ideas. To some extent, leadership behavior can represent the position of the organization [29]. Therefore, according to the social exchange theory, employees will make some extra-role behaviors in return for the positive feedback of leaders and organizations. Safety voice is a kind of extra-role behavior. Whether voice is made or not depends on the individual will of employees and enterprises cannot force employees to voice through rules and regulations [47]. In addition, safety voice is a kind of behavior to avoid personal injury in a dangerous environment. It can identify the risks in the organization and avoid the disastrous consequences, which is conducive to the long-term development of the enterprise. Therefore, employees may repay the organization through safety voice.

LMX is a differentiated dynamic interaction between leaders and subordinates [48,49]. Leaders will allocate resources based on their relationship with subordinates. Those who have a higher quality exchange relationship with leaders will get more work resources and have a higher enthusiasm for work [15]. Social exchange theory emphasizes that exchange subjects should follow the principles of reciprocity and fairness in the exchange process. The principle of reciprocity means that the exchange activity is not a one-sided give or take, but both parties will get a return, and both parties will also pay a certain cost, otherwise social interaction will not last or even not happen. Therefore, a high-quality LMX relationship will improve employees’ work attitude and organizational citizenship behavior [50]; many studies have found that LMX relationship can promote employee voice [15,51].

There are certain risks that may prevent employee safety voice [52,53]. Leaders in a high-quality LMX relationship may give employees more safety support. When employees feel the safety support from the leaders, they will think that the risk of speaking out about safety concerns and suggestions are relatively small [54], so they are more likely to make safety voice. Previous studies have demonstrated the mediating role of LMX between supportive leadership behaviors (e.g., authentic leadership) and employee voice behavior [37,55,56]. Accordingly, the following hypothesis is proposed:

**Hypothesis** **4.** *LMX plays a mediating role in the relationship between voice endorsement and the employee safety voice*.

**Figure 1:**
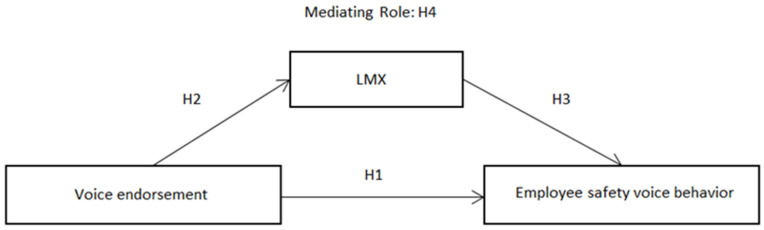
Research hypothesis model.

This study is based on social exchange theory, taking voice endorsement as an independent variable, employee safety voice behavior as a result variable, and introducing LMX as an mediating variable to build a research hypothesis model, as shown in Figure 1.

## 3. Materials and Methods

### 3.1. Sample and Procedure

The sample participants were workers from three large construction companies in Hebei, China. To eliminate the common method bias [57], first, we emphasized in the guidance of the questionnaires that this was an anonymous survey, and there was no right or wrong answer. A nested questionnaire was used to distribute the large envelope containing the questionnaire to the leaders, who evaluated the members of the organization and sent the small envelope inside the big envelope to the subordinate employees to fill in. The small envelope contained one to two employee self-assessment questionnaires, which were filled in by employees. Among them, the questionnaires filled in by leaders included voice endorsement, and employee self-assessment questionnaires included employee safety voice behavior and LMX. All completed questionnaires were sealed in envelopes and matched with codes. Second, we used a three-wave, time-lagged multi-sourced data collection method to obtain the leader-employee matching questionnaires. The survey was administered in September 2019 for the first company, October 2019 for the second company, and November 2019 for the third company. We first distributed the leader questionnaires to the leaders of the companies and then distributed the employee questionnaires to the direct subordinates through their respective leaders at an interval of 2 weeks. The questionnaires of leaders and employees were collected 5 days after the questionnaires were issued.

We received 671 responses. After deleting questionnaires with severely missing information, concentrated answers, or obvious regularity, our final sample consisted of 558 valid leader-employee matching responses with an overall response rate of 83.16%. Among them, 214 were leaders, 133 were male, accounting for 62.15%; 134 were aged 26–35, accounting for 62.62%; 189 had a bachelor degree or above, accounting for 88.32% of the total sample; 189 had worked for 3–7 years, accounting for 41.59% of the total sample. There were 344 employees in the sample, 215 were female, accounting for 62.50%; 148 were aged 26–35, accounting for 43.02%; 262 had bachelor’s degree or above, accounting for 76.16% of the total sample; 114 employees had worked for 1–3 years, accounting for 33.14% of the total sample.

### 3.2. Measures

This study mainly discusses the influence of voice endorsement on employee safety voice behavior and adds LMX as a mediating variable. The scales used in this study were mature scales developed by scholars. For the items whose original scale was English, we used double-blind translation and back translation to ensure semantic equivalence after translation [58]. To adapt to the specific situation of this study, based on keeping the original meaning of the items unchanged, we made necessary adjustments to the expression of some items, trying to make the expression after adjustment simple and easy to understand, without ambiguity, and in line with the expression habits of Chinese. Likert 5-point score was used for all variables except demographic variables. A score of 1 indicated total disagreement and 5 indicated full agreement, forming the questionnaire. Table A1 shows the survey items used in this paper with their authors and article sources.

#### 3.2.1. Voice Endorsement

We measured voice endorsement using Burris [9]’s five-item scale. Typical items were “I think this subordinate’s comments should be implemented” and “I will take this subordinate’s comments to my supervisors”. In this study, the Cronbach’s α value of the scale was 0.898.

#### 3.2.2. LMX

We used a seven-item scale developed by Graen and Uhl-Bien [21] to measure LMX. The representative items were “I know how satisfied my leader is with what I do”, “My leader understands my job problems and needs” and “Regardless of how much formal authority he/she has built into his/her position, I think my leader would use his/her power to help me solve problems in my work”, which were filled in by employees. In this study, Cronbach’s α was 0.913.

#### 3.2.3. Employee Safety Voice

We used a five-item scale designed by Tucker and Chmiel [3] to measure employee safety voice behavior. Sample items include: “I can make suggestions to improve work safety” and “I discuss new ways to improve safe working with my colleagues or boss”, which were filled in by employees. In this study, Cronbach’s α value of the scale was 0.861.

#### 3.2.4. Control Variables

Prior research showed that the result variable employee safety voice behavior was affected by gender, age, and other control variables [7,59]. Therefore, this study took employees’ gender, age, education level, and work experience as control variables. We also measured leaders’ gender, age, education level, and work experience.

## 4. Data Analysis and Results

### 4.1. Homology Analysis of Variance

Subjective factors of the subjects may cause homology bias in the measured variables. In this study, the Harman single factor method was used. The principal component analysis of unrotated factors was performed in SPSS 22.0 and the variance contribution rate of the first factor was extracted to test whether the homology deviation exists. Analysis of the unrotated principal component factors showed that the cumulative variance explained by the first factor is 39.810%, which was less than 40%. It indicates that a single factor could not explain most of the variance [57].

### 4.2. Confirmatory Factor Analysis

The discriminative validity test was performed by confirmatory factor analysis using SPSS 22.0. The measurement model had three factors, including voice endorsement, LMX, and employee safety voice behavior. The measurement results are shown in Table 1. The results show that the three-factor model fits well ($\chi^{2}/df$ = 1.104, GFI = 0.974, NFI = 0.973, CFI = 0.997, TLI = 0.997, RMSEA = 0.014), and it is significantly better than the two-factor model A ($\chi^{2}/df$, GFI = 0.791, NFI = 0.825, CFI = 0.846, TLI = 0.822, RMSEA = 0.089), two-factor model B ($\chi^{2}/df$ = 8.629, GFI = 0.745, NFI = 0.784, CFI = 0.803, TLI = 0.773, RMSEA = 0.099), two-factor model C ($\chi^{2}/df$ = 7.660, GFI = 0.762, NFI = 0.808, CFI = 0.828, TLI = 0.802, RMSEA = 0.096), and single-factor model ($\chi^{2}/df$ = 14.191, GFI = 0.742, NFI = 0.766, CFI = 0778, TLI = 0.728, RMSEA = 0.119), which shows that the discriminative validity of each scale is good and can be analyzed in the next step.

### 4.3. Descriptive Statistical Analysis and Correlation Analysis

Table 2 shows the mean, standard deviation, and correlation coefficient of each variable. Among them, voice endorsement was negatively correlated with employee safety voice behavior (r = −0.381, *p* < 0.01), and voice endorsement was negatively correlated with LMX (r = −0.460, *p* < 0.01); LMX was positively correlated with employee safety voice behavior (r = 0.437, *p* < 0.01).

### 4.4. Test of Hypotheses

In this study, a multi-level linear model was used for hypothesis testing, and the model test was completed in three steps. In the first step, the control variables such as gender, age, education level, and work experience of employees are substituted into the model; the second step is to verify the direct effect of voice endorsement on employee safety voice behavior; the third step is to verify the indirect effect of voice endorsement on LMX and LMX on employee safety voice behavior.

The regression analysis results are shown in Table 3. Take the control variables (gender, age, education level, and work experience) as independent variables, and employee safety voice behavior as dependent variables, and establish model 3. It can be seen from Table 3 that gender has no significant effect on employee safety voice behavior, age positively affects employee voice behavior (model 3, β = 0.145, *p* < 0.01), education level positively affects employee safety voice behavior (model 3, β = 0.522, *p* < 0.01), and work experience has no significant effect on the employees safety voice behavior. Voice endorsement is introduced as an independent variable into model 3, and model 4 is established to test the main effects. It can be seen from Table 3 that voice endorsement has a significantly negative impact on employee safety voice (model 4, β = −0.384, *p* < 0.01).

The control variables (gender, age, education level, and work experience) are used as independent variables, and LMX is used as the dependent variable to establish model 1. It can be seen that gender, age, and educational level have no significant influence on the LMX, while work experience has a positive impact on LMX (model 1, β = 3.912, *p* < 0.01).

The results of regression analysis are shown in Table 3. As for the main effect test, it can be seen from the table that voice endorsement has a significantly negative impact on employee safety voice behavior (model 4, β = −0.384, *p* < 0.01).

As for the indirect effect test, it can be seen that voice endorsement has a significantly negative impact on LMX (model 2, β = −0.542, *p* < 0.01); LMX has a significantly positive effect on employee safety voice behavior (model 5, β = 0.362, *p* < 0.01).

In order to test the mediating effect of LMX, we use the Monte Carlo method to conduct a Bootstrapping test on the mediating effect of LMX, as shown in Table 4.

Table 4 shows the size, standard error, and 95% confidence interval of indirect effects. The results show that the indirect effect of LMX is significant in the path of voice endorsement, inhibiting employee safety voice behavior. Moreover, both of them do not contain 0 in the 95% confidence interval [−0.232, −0.129] for a repeated sampling of 1000 times, which indicates that LMX played a mediating role between voice endorsement and employee safety voice behavior, which further verified Hypothesis 4. Table 5 shows the hypothesis validation results.

Only when the independent variables are exogenous, the linear regression model can test the causal relationship between the variables [60]. Questionnaire survey data may have endogeneity problems due to the difficulty of satisfying strict exogeneity [61]. To solve the endogeneity problem, we use Two Stage Least Squares (2SLS) [62]. We used the gender and age of the leaders as instrumental variables, because the leaders’ characteristics affect leaders’ voice endorsement, but do not directly affect employee safety voice behavior. In addition, we took voice endorsement as the endogenous variable, safety voice as the explanatory variable, and the mediating variable—LMX—as the exogenous variable [63].

Table 6 shows the results of the 2SLS test. As can be seen from the above table, $R^{2}$ of the model is 0.162, which means that the voice endorsement and LMX can explain the 16.2% change of safety voice. When the Wald chi square test was conducted on the model, it was found that the model passed the Wald chi square test ($\chi^{2}$ = 135.448, *p* = 0.000 < 0.05), which means that at least one of voice endorsement and LMX will have an impact on the safety voice. In conclusion, LMX produces a significant positive influence relationship on safety voice (β = 0.188, *p* < 0.05), as well as voice endorsement produces a significant negative influence relationship on safety voice (β = −0.606, *p* < 0.01).

The Durbin–Wu–Hausman test is used to test whether the explanatory variables X are all exogenous. As can be seen from Table 7, *p* < 0.05, it means that not all explanatory variables are exogenous, i.e., the explanatory variable X contains endogenous variables that satisfy the premise of the instrumental variables approach.

The overidentifying test was used to test whether the instrumental variables were exogenous or not, and this study involved two instrumental variables, which were the gender and age of the leaders. As can be seen from Table 8, the Sargan test shows acceptance of the original hypothesis (*p* = 0.195 > 0.05), while the Basmann test also shows acceptance of the original hypothesis (*p* = 0.196 > 0.05). The same indicates that the assumption of ‘exogeneity of instrumental variables’ cannot be rejected and the model is good.

## 5. Discussion

This section first discusses the results of data analysis, then points out the theoretical and practical implications of this article, and finally summarizes the deficiencies in the full text of the research process and the prospects for such research in the future.

### 5.1. Discussion of Results

The results of multi-level regression analysis show that voice endorsement has a significantly negative effect on employee safety voice in construction projects. Tucker and Chmiel [3] pointed out that safety voice proposes behaviors intended to avoid deficiencies in the work environment or improve working environment conditions to specific objects. This definition clearly states that implementing safety advice requires specific measures to change the current work status, thereby improving safety in the workplace. Safety voice mainly expresses the consideration of safety performance. Its improvement is often not related to the improvement of product performance in the short term. It may even lead to the temporary weakening of production performance due to changes in current production methods [64,65]. Workers in Chinese construction companies often violate safety regulations to save time and energy at work, which brings safety hazards [66]. Voice endorsement can reduce and eliminate these hidden safety hazards. Still at the same time it will also lead to more complex work processes and longer working hours. On the one hand, workers will not make safety voice for their own convenience; on the other hand, the inconvenience caused by voice endorsement will also harm the interests of the proponent’s colleagues and cause tension in interpersonal relationships. Therefore, employees will give up safety voice under pressure from colleagues. Previous studies have also confirmed that colleagues’ attitudes towards work safety have an important influence on employee safety voice [3].

In addition, the analysis results show that voice endorsement has a significantly negative effect on LMX in construction projects. In the research on the antecedents of employee voice behavior, leaders are regarded as the key factor that stimulates or inhibits employee voice [67]. Voice endorsement refers to the degree of concern and acceptance of the suggestions and opinions put forward by subordinates by managers [68]. Leaders’ voice endorsement means that the leaders agree with the ideas of the employees. However, some studies have shown that leaders may deliberately refuse to adopt reasonable employee voice because they are worried that the dissatisfaction implied in employee voice will threaten their image and status [9,69,70]. In this study, employee safety voice may not be treated correctly by the leaders. However, when the leaders do not pay attention to the quality of employee safety voice, they frequently adopt employee safety voice or often adopt unreasonable safety voice, which will make employees question the ability of leaders to deal with suggestions and reduce the communication with leaders. The quality of LMX is affected by the quality and frequency of communication between leaders and subordinates. So when the communication frequency between employees and leaders is reduced, the quality of LMX will also decrease [71]. Also, as a supportive resource, voice endorsement can easily cause emotional exhaustion in employees due to excessive pressure to ask for rewards. Individuals perceive a state of chronic fatigue after overuse of energy, with signs of low energy and loss of interest in things, which is emotional exhaustion [72]. Emotional exhaustion is a consequence of excessive stress on employees in the workplace and can lead to a decrease in employees’ commitment to work operations and a willingness to disengage from the organization and reduce communication exchanges with leaders [73]. The quality of LMX is influenced by the quality and frequency of interactions between leaders and subordinates [71], and when employees communicate with their leaders less frequently, the quality of LMX also decreases.

This study also finds that in construction projects LMX plays a mediating role between voice endorsement and employee safety voice behavior. The samples in this study are all construction workers, their work places are construction sites, their leaders work in the office, and they do not have a full understanding of the construction site conditions, so they may endorse unreasonable voice. Therefore, unreasonable voice may be endorsed, which may cause employees to question the leaders’ ability. Employees who are not convinced of the leadership’s ability will consciously reduce the frequency of communication with the leader, thereby reducing the quality of LMX between both parties [69,71].

Graen and Uhl-Bien [21] argued that the attitude and supportive behavior of leaders to employees are greatly affected by the quality of LMX. When there is a high quality of LMX between employees and leaders, leaders will provide employees with more work resources. Employees will take certain risks in the process of safety voice. The trust and support of leaders to employees will reduce employees’ risk perception, thus increasing the possibility of safety voice [37]. According to social exchange theory, when employees get extra support from leaders and organizations, they will have a sense of obligation to repay the organization [22]. It is also more likely to make safety voice for improving organizational safety. Therefore, voice endorsement will negatively affect LMX and then reduce the safety voice behavior of employees. LMX plays a mediating role between voice endorsement and employee safety voice.

### 5.2. Implications

This research has both theoretical and practical implications.

#### 5.2.1. Theoretical Implications

First, from the theoretical and empirical aspects, we analyzed and inspected the effect and mechanism of the leaders’ suggestions on employee safety voice behavior, thus perfecting the theoretical understanding of the employee safety voice formation mechanism. In previous studies, scholars focused on the impact of leadership on employee safety voice [6,7]. Relatively speaking, these leadership styles are more of an environmental factor for safety voice behavior. Leaders’ voice endorsement has more direct significance for safety voice and is the key link for safety voice to achieve its purpose. Based on the perspective of social exchange, this study reveals the inhibitory effect of voice endorsement on employees. Some unsafe behaviors of employees can save working time and physical strength, and the measures taken by leaders after adopting safety voice will reduce the work efficiency of workers [66]. Therefore, workers are not willing to make safety voice to protect their interests. Therefore, based on previous studies, this article provides new theoretical logic and empirical evidence for analyzing the incentives of voice endorsement and also enriches the theoretical research on employee safety voice.

Second, for the first time, LMX was included in the research framework for voice endorsement to affect employee safety voice. The study found that the relationship between LMX in voice endorsement and employee safety voice, that is, voice endorsement negatively affects employee safety voice behavior by negatively affecting LMX. While some previous studies have shown that supportive leadership behaviors promote LMX [39,56], the results of this paper indicate that voice endorsement can negatively affect LMX, which provides new ideas for LMX theory research. In addition, in the past, scholars mostly used LMX as the antecedent variable of employee voice [5,15,51], seldom examined its impact on safety voice, and this study verifies that LMX promotes employee safety voice behavior and its negatively mediating role between voice endorsement and employee safety voice behavior. Therefore, this study expands the exploration of the influence of LMX on employee behavior.

Third, based on the perspective of equality and reciprocity in the social exchange theory, this study explains the internal mechanism of voice endorsement and employee safety voice behavior and finds that voice endorsement can actually inhibit employee safety voice behavior. In the past, there were few literatures on safety voice behavior based on the perspective of social exchange, and most discussions on safety voice based on social exchange theory only considered the factors of leadership style [3,6,7], while ignoring the specific behavior of leaders such as voice endorsement. Therefore, this study provides a new idea for exploring employee safety voice behavior based on social exchange theory.

Fourth, employee voice started out as collective voice, but gradually shifted to individual voice due to the weakening of the union [74,75]. Although less attention has been paid to collective employee voice in the current literature, collective employee voice still plays an important role in organizations [76], for example, collective employee voice is closely related to “capacity to aspire” and “social identity” [77,78]. Safety voice discussed in this paper refer to individual voice. Individual and collective contributions are strongly linked, and they both contribute to organizational performance and can work synergistically to play a positive role in the organization [76,79]. Therefore, we suggest that the conclusions of this paper on individual voice are important references for collective voice.

#### 5.2.2. Practical Implications

The research results of this study help managers understand the formation mechanism of employee safety voice behavior and then provide reference opinions for promoting employee safety voice behavior.

First of all, the research results show that in construction projects, voice endorsement has a significantly negative effect on LMX and employee safety voice. This suggests that managers must improve the employee safety voice system to form a good atmosphere for everyone to proactively make safety voice. Employee safety voice content should take a cautious attitude, not blindly adopt low-quality safety voice, and let employees form a belief that only high-quality safety voice will cause leader’ attention and adoption.

Second, after the above research in this paper, it is found that the LMX has a significantly positive effect on employee safety voice in construction projects. It can be seen that in enterprises, the establishment of positive leadership employee relations can promote employee safety voice and further improve enterprise safety performance. To establish a good leadership and employee relationship, on the one hand, we must take the initiative to observe the work of employees and give encouragement and appropriate help when employees encounter difficulties; on the other hand, we must learn to respect and trust employees and form a mutually beneficial work partner. The relationship enables employees to voluntarily fulfill their responsibilities for the development of the organization and to make efforts beyond the responsibilities to put forward their own opinions and suggestions on issues in the organization.

### 5.3. Limitations and Future Research Directions

First of all, the mediating variable and dependent variable involved in this research came from employee self-evaluation, which may lead to homology variance [57], and affected the research results. Therefore, in the follow-up study, researchers can try to measure safety voice behavior from different subjects, including employees, their leaders, and colleagues, and get more objective evaluation results through integration and comparison.

Secondly, the research object of this paper was Chinese construction workers. Although the employee safety voice behavior, voice endorsement, and LMX scale are mature scales widely used in related research, they were not developed based on the Chinese context [6,9]. Therefore, with many scholars trying to measure the above variables in Chinese culture, it is necessary to develop appropriate scales according to the characteristics of different studies.

Thirdly, this paper only considered the mediating role of LMX when examining the mechanism of voice endorsement on employee safety voice. Voice endorsement may also affect employee safety voice behavior through other paths and boundary conditions, so future research should try to introduce other mediating variables or moderating variables to explain the mechanism of voice endorsement on employee safety voice behavior.

Finally, this paper did not take into account organizational features, which may have affected the accuracy of the results, as organizational features can affect employee behavior [31]. Therefore, future studies should consider not only independent and dependent variables, but also organizational features, such as safety climate.

## 6. Conclusions

Based on the social exchange theory, this research discusses the impact of voice endorsement on employee safety voice and its impact mechanism in construction projects, focusing on the mediating role of LMX. The results show that in construction projects, voice endorsement has a significantly negative impact on employee safety voice behavior, and at the same time verifies the negative mediating role of LMX between voice endorsement and employee safety voice behavior. Voice endorsement has a negative effect on employee safety voice behavior through negative influence of LMX. The results of this study can provide a theoretical basis for the future leadership behavior’s impact mechanism on employee safety voice behavior. Future research can also be conducted from other theoretical perspectives, such as the theory of planned behavior, to study employee safety voice behavior. In addition, the research object of this paper is workers in the construction industry, and future research can focus on the safety voice behavior of employees in other industries. Finally, this paper only discusses the mediating role of LMX between voice endorsement and employee safety voice behavior, and many studies currently use leadership behaviors as antecedents of LMX, and fewer studies have explored the effects of LMX on leadership behavior, so future attention could also be paid to how LMX acts on voice endorsement.

## Figures and Tables

**Table 1:** Results of confirmatory factor analysis of study variables.

Model	Factor	$\chi^{2}/df$	GFI	NFI	CFI	TLI	RMSEA
Three-factor	VE, LMX, SV	1.104	0.974	0.973	0.997	0.997	0.014
Two-factor A	VE + LMX, SV	6.989	0.791	0.825	0.846	0.822	0.089
Two-factor B	VE, LMX + SV	8.629	0.745	0.784	0.803	0.773	0.099
Two-factor C	VE + SV, LMX	7.660	0.762	0.808	0.828	0.802	0.096
One-factor	VE + LMX + SV	14.191	0.742	0.766	0.778	0.728	0.119

Note: *n* = 558; VE = voice endorsement, LMX = leader member exchange, ESV = employee safety voice; “+” represents the combination of the two factors.

**Table 2:** Descriptive statistics, reliabilities, and correlations.

Variable	M	SD	1	2	3	4	5	6	7	8	9	10	11
1. Gender	1.643	0.479	1										
2. Age	1.858	0.806	0.171 **	1									
3. EL	2.776	0.669	0.109 *	0.184	1								
4. WE	2.333	1.012	−0.014	0.443 **	0.153 *	1							
5. Gender (Leader)	1.379	0.486	0.257	0.213 *	0.421	0.211	1						
6. Age (Leader)	2.318	0.599	0.366	0.017	0.303 **	0.040	−0.028	1					
7. EL (Leader)	2.991	0.597	0.268	0.235 *	0.295 *	0.192	0.093	0.008	1				
8. WE (Leader)	2.724	0.824	0.366	0.225 *	0.487	0.268	−0.055	0.052 **	−0.110	1			
9. VE	3.970	0.616	−0.244 **	−0.138 **	−0.085 *	−0.067	−0.037	0.010	0.182 **	0.054	1		
10. LMX	3.574	0.736	0.085 *	0.128 **	0.105 *	0.201 **	−0.460 **	0.030	0.159 **	0.010	0.193 **	1	
11. ESV	3.776	0.710	0.102 *	0.274 **	0.529 **	0.182 **	−0.381 **	0.437 **	−0.111 *	0.059	−0.012	0.130 *	1

Note: * *p* < 0.05, ** *p* < 0.01, EL = Educational level, WE = Work experience, VE = voice endorsement, LMX = leader-member exchange, ESV = employee safety voice. Later same.

**Table 3:** Regression analysis results for voice endorsement, LMX, and employee safety voice behavior.

Variable	LMX		Employee Safety Voice Behavior		
	Model 1	Model 2	Model 3	Model 4	Model 5
Gender	0.117	−0.040	0.030	−0.081 **	−0.012
Age	0.020	−0.013	0.145 **	0.122 **	0.318 **
EL	0.072	0.051	0.522 **	0.508 **	0.496 **
WE	0.912 **	0.123 **	0.024	0.017	−0.024
Gender (Leader)	0.057	0.081	−0.127	−0.139 **	−0.161 **
Age (Leader)	0.050	0.069	−0.029	−0.019	−0.059
EL (Leader)	0.037	0.042 **	0.004	−0.009	−0.018
WE (Leader)	0.103 *	0.133	0.088 *	0.073 *	0.027
VE		−0.542 **		−0.384 **	
LMX					0.362 **
*R* ^2^	0.053	0.244	0.053	0.314	0.447
Δ*R*^2^	0.053	0.191	0.053	0.314	0.134
F	7.714 ***	35.580 ***	7.714 ***	63.170 ***	133.314 ***

Notes: * *p* < 0.05, ** *p* < 0.01, *** *p* < 0.001.

**Table 4:** Test results of mediating effect based on LMX.

Items	Effect	Boot SE	BootLLCI	BootULCI	z	*p*
VE⇒LMX⇒SV	−0.176	0.027	−0.232	−0.129	−6.557	0.000

Note: BootLLCI refers to the lower limit of 95% interval of bootstrap sampling, and BootULCI refers to the upper limit of 95% interval of bootstrap sampling.

**Table 5:** Hypothesis testing results.

Hypothesis	Hypothesis Validity (Yes/No)
H1: Voice endorsement positively influences employee safety voice behavior	No
H2: Voice endorsement positively influences LMX	No
H3: LMX positively influences employee safety voice behavior	Yes
H4: LMX plays a mediating role in the relationship between voice endorsement and the employee safety voice	Yes

**Table 6:** 2SLS model analysis results.

Variable	Employee Safety Voice
Voice Endorsement	−0.606 **
LMX	0.188 *
*R* ^2^	0.162
Δ*R*^2^	0.159
Wald $\chi^{2}$	$\chi^{2}\left(2\right)$ = 135.448, *p* = 0.000

Notes: * *p* < 0.05, ** *p* < 0.01.

**Table 7:** Test of exogeneity.

Test Method	Hypothesis	Test Results	Test Conclusion
Durbin Test	All explanatory variables are exogenous	$\chi^{2}\left(1\right)$ = 4.722, *p* = 0.030	Rejecting the hypothesis
Wu-Hausman Test	All explanatory variables are exogenous	F(1554) = 4.728, *p* = 0.030	Rejecting the hypothesis

**Table 8:** Overidentifying restrictions.

Test Method	Hypothesis	Test Results	Test Conclusion
Sargan Test	All instrumental variables are exogenous	$\chi^{2}\left(1\right)$ = 1.678, *p* = 0.195	Accepting the hypothesis
Basmann Test	All instrumental variables are exogenous	$\chi^{2}\left(1\right)$ = 1.671, *p* = 0.196	Accepting the hypothesis

## Data Availability

The data presented in this study are available on request from the corresponding author.

## Appendix A

**Table A1:** Survey items and sources.

Variables	Items	Authors and Articles
Voice Endorsement	1. I will take this subordinate’s comments to my supervisors. 2. I will support this subordinate’s comments when talking with my supervisors. 3. I think this subordinate’s comments should be implemented. 4. I agree with this subordinate’s comments. 5. This subordinate’s comments are valuable.	Burris (2012) The risks and rewards of speaking up: Managerial responses to employee voice
LMX	1. I know how satisfied my leader is with what I do. 2. My leader understands my job problems and needs. 3. My leader can recognize my potential. 4. Regardless of how much formal authority he/she has built into his/her position, I think my leader would use his/her power to help me solve problems in my work. 5. Regardless of the amount of formal authority my leader has, I think he/she would “bail me out,” at his/her expense. 6. I have enough confidence in my leader that I would defend and justify his/her decision if he/she were not present to do so. 7. I have a friendly relationship with my leader at work.	Graen, Uhl-Bien (1995) Relationship-based approach to leadership: Development of leader-member exchange (LMX) theory of leadership over 25 years: Applying a multi-level multi-domain perspective
Employee Safety Voice	1. I make suggestions about how safety can be improved. 2. I tell my colleague who is doing something unsafe to stop. 3. I discuss new ways to improve safe working with my colleagues or boss. 4. I inform the union/boss when I notice a potential driving hazard. 5. I report to my boss if my colleagues break any safety rules.	Tucker et al. (2008) Perceived organizational support for safety and employee safety voice: the mediating role of coworker support for safety
